# IMPLEMENTATION OF EVIDENCE-BASED INTERVENTIONS ACCORDING TO THE SWEDISH NATIONAL GUIDELINES FOR STROKECARE: A NATIONWIDE SURVEY AMONG PHYSIOTHERAPISTS

**DOI:** 10.2340/jrm.v56.18444

**Published:** 2024-03-19

**Authors:** Sara BRYCKE, Anna BRÅNDAL, Christina BROGÅRDH

**Affiliations:** 1 Department of Neurology, Rehabilitation Medicine, Memory Disorders and Geriatrics, Skåne University Hospital, Lund; 2 Department of Community Medicine and Rehabilitation, Physiotherapy, Umeå University, Umeå; 3 Department of Health Sciences, Lund University, Lund, Sweden

**Keywords:** rehabilitation, stroke, national guidelines, physiotherapy, implementation

## Abstract

**Objective:**

To investigate (*i*) to what extent physiotherapists (PTs) working in stroke rehabilitation in various parts of the stroke care chain have implemented interventions according to the national guidelines for stroke (NGS), (*ii*) facilitating and hindering factors for the implementation, and (*iii*) differences between various care settings.

**Design:**

A cross-sectional study.

**Subjects:**

148 PTs working in stroke rehabilitation in various parts of the care chain in Sweden.

**Methods:**

Data were collected by a web-based survey.

**Results:**

Task-specific training for walking (80–98%), impaired motor function (64–100%) and fall prevention (73–92%) were most implemented. Factors that facilitated implementation were: important to comply with the NGS, that PTs had confidence to perform the interventions, and that interventions were clearly described. Limited time, lack of resources, no clear goals or routines at the workplace hindered the implementation. Significant differences (*p* < 0.05) between the settings existed. Municipal and primary care reported most challenges in implementing the NGS and providing evidence-based interventions.

**Conclusion:**

Most interventions, with high priority according to NGS, are provided by PTs working in stroke rehabilitation, although differences in various parts of the care chain exist. Knowledge, time, education and supportive management are important factors when implementing evidence-based interventions.

LAY ABSTRACT

To facilitate care and rehabilitation after stroke becoming more nationally aligned, the National Board of Health and Welfare regularly updates national guidelines for stroke. Currently, there is a lack of knowledge regarding the extent to which physiotherapists working in stroke rehabilitation have been able to implement the guidelines. In this study, 148 physiotherapists working in stroke rehabilitation in various parts of the care chain responded to a digital survey with questions on which rehabilitation interventions had been implement-ed, and factors that have facilitated or hindered the process. We found that most recommendations, with high priority according to the national guide-ines for stroke, were implemented to a great extent, although there were differences between care settings. Knowledge, time, education and supportive management are important for successful implementation of the guidelines. These findings could be used to improve the implementation process of evidence-based rehabilitation interventions throughout the stroke care chain.

Stroke is one of the most common causes of disability in the adult population in Sweden and worldwide (1). In Sweden, approximately 25,000 individuals suffer a stroke every year (1). As primary prevention and acute medical treatments have improv-ed at stroke onset, an increased number of people living with remaining disabilities following stroke is expected (1–3). A multidisciplinary team with good knowledge of stroke rehabilitation can improve recovery and outcomes after stroke (2, 4–6). Today, access to rehabilitation after stroke is, however, unequally distributed in Sweden (7).

The National Board of Health and Welfare regularly updates national guidelines for stroke (NGS), to provide recommendations on how the healthcare system should prioritize its resources to provide as efficient and equal care and rehabilitation as possible (1). The latest version was updated in 2018/2019 (1). However, although there is evidence for several rehabilitation interventions after stroke, they can be difficult to implement in clinical practice (8–11). A theoretical framework that can be used to facilitate the implementation process is the PARIHS model (Promoting Action on Research Implementation in Health Services) (9). The three most important factors when implementing new knowledge, according to this model, are: characteristics of the *evidence*, the *context* (such as culture, leadership and evaluation), and the *facilitation*, i.e. the process making implementation easier (9). It is the interaction between these factors that determines how successful the implementation will be (9–11). Previous studies have reported that lack of resources, routines, support from managers, insufficient training and knowledge on how to use the guidelines are barriers to implementing guidelines and evidence-based interventions (12–15).

Currently, there are geographical differences in Sweden regarding access to stroke rehabilitation and the type of interventions provided after discharge from hospitals (7). Three months after stroke onset the proportion of people who are satisfied with their rehabilitation is lower than those who are satisfied with their healthcare in general (7). Several studies have also described that many stroke survivors are dissatisfied with access to their rehabilitation needs (5, 16–19). These findings underscore the necessity to explore avenues to successfully facilitate the implementation of guidelines in clinical settings to contribute to more equitable care and rehabilitation after stroke across the country.

Physiotherapists (PTs) play an important role in the multidisciplinary team that works in stroke rehabilitation (20–22). Many of the interventions that PTs provide have been given high priority in the NGS (1). However, there is a lack of knowledge concerning the extent to which PTs in various parts of the care chain in Sweden (i.e. stroke units, hospital in- and outpatient rehabilitation, primary care and municipal care), have been able to implement the NGS. Increased knowledge can contribute to improve the implementation of evidence-based rehabilitation interventions (12). The aim of this study was therefore to investigate (*i*) to what extent PTs who work in stroke rehabilitation in various part of the stroke care chain in Sweden have implemented the NGS; (*ii*) which factors have facilitated/hindered the implementation process and (*iii*) differences between the care settings.

## METHODS

### Study design

This study has a cross-sectional design, and is based on a digital web-based survey. When reporting the data, the STROBE checklist for cross-sectional studies was followed.

### Recruitment of participants

The digital survey was distributed to PTs working in stroke rehabilitation in various settings in Sweden, such as stroke units, hospital in- and outpatient rehabilitation, primary care and municipal care (in- and outpatient rehabilitation, home-based rehabilitation and nursing homes). Via the Swedish Association of Physiotherapists, an information letter and a link to the survey were directed to PTs specialized in neurology. Also, via managers working in stroke rehabilitation at hospitals, primary care centres and municipalities, brief information about the study, an information letter and a link to the survey were e-mailed to PTs at their departments. The inclusion criterion for participation was working in stroke rehabilitation as a PT in various levels of the care chain in Sweden.

### Data collection

The digital survey was constructed in Microsoft Forms with questions inspired by the PARIHS model (i.e. on evidence, context and facilitation of implementation) (9), and by previous studies on factors that have facilitated or hindered the implementation of NGS (10–13, 23–25). Thus, the survey comprised background information, questions about the extent to which the PTs provide evidence-based rehabilitation interventions recommended in the NGS (priority 1–7, see below), and questions about factors that have facilitated/hindered the implementation process at the workplace in the different care settings. The recommendations in the NGS (1) range from 1 to 10: interventions that health care *should provide* (priority 1–3), interventions that health care *may provide* (priority 4–7) and interventions that health care *may provide in exceptional cases* (priority 8–10). The NGS also comprises interventions that health care should not perform routinely unless in research and also interventions that should not be provided at all (1).

In total, the survey comprised 59 closed questions. The questions related to the interventions in the NGS had five response options (always provided, frequently provided, rarely provided, not provided, not relevant in my clinical setting), but it was also possible to add information in free text. To ensure that the questions in the survey were not misinterpreted, a pilot survey was distributed to 7 PTs involved at various levels of the stroke care chain prior to the study start. Based on their comments, slight adjustments were made in the final version. The survey was distributed to PTs between 14 January and 18 March 2022, and took 15–20 min to complete.

### Data analysis

Data were compiled and analysed in IBM SPSS Statistic version 25 (IBM Corp, Armonk, New York, USA). Descriptive statistics *n* (%) were used to characterize the participants’ demographics, rehabilitation interventions provided, and perceived factors that had facilitated/hindered the implementation process. In the analysis, the five response options in the survey were pooled into three: “always/ frequently provided”, “rarely/not provided”, “not relevant in my clinical setting” to simplify the presentation of the responses.

The Kruskal–Wallis test was used to analyse whether there were any statistically significant differences between the settings (i.e. stroke unit, hospital in- and outpatient rehabilitation, primary care, municipal care) regarding number of interventions provided, and perceived factors that facilitated and hindered the implementation of NGS. Thereafter, pairwise post-hoc tests were performed to reveal between which settings significant differences existed. Significance levels (*p* < 0.05) were adjusted by the Bonferroni correction for multiple tests.

### Ethics

All individuals gave written informed consent to participate in the study. An ethical application was sent to the Swedish Ethical Review Authority. However, because the project did not involve any intervention on a research subject or include personal data, the Ethical Review Authority did not consider that the study needed ethical review, but had no ethical objections to the project (Dnr 2022-00899-01). The study was conducted in accordance with the Declaration of Helsinki.

## RESULTS

In total, 148 PTs participated in the study (Table I). Most of them were women (82%). They worked at various levels within the care chain although hospital in- and outpatient rehabilitation was most common (35%). More than half (55%) had further education in neurological rehabilitation, but only a few (9%) were specialists in neurology. Completed answers were returned from all 21 counties in Sweden and from 19 of 290 municipalities.

**Table I:** Descriptive characteristics of the participants (*n* = 148)

Age groups	*n* (%)
20–29 years	28 (19)
30–39 years	46 (31)
40–49 years	36 (24)
50–59 years	30 (20)
60–69 years	8 (5)
Gender (women)	122 (82)
Years working as physiotherapist	
0–4	25 (17)
5–9	27 (18)
10–14	22 (15)
15–19	31 (21)
> 20	43 (29)
Years of experience in stroke rehabilitation	
0–4	48 (32)
5–9	24 (16)
10–14	26 (18)
15–19	23 (16)
> 20	27 (18)
Further education in stroke rehabilitation	
Yes	82 (55)
No	66 (45)
Specialist in neurology	
Yes	14 (9)
No	134 (91)
Care chain levels	
Stroke unit	28 (19)
Hospital in- and outpatient rehabilitation	51 (35)
Primary care	30 (20)
Municipal care	39 (26)

### Interventions that always/frequently were provided

Interventions that a majority of the PTs reported as always/frequently provided were: task-specific gait training (80–98%), fall prevention (73–92%) and task-specific training due to impaired motor function (64–100%). Almost three-quarters of the PTs working in a stroke unit or an early supported discharge (ESD) team reported that ESD and continued rehabilitation at home were always/frequently provided. Of the PTs working in hospital in- and outpatient rehabilitation and in primary health care, a majority reported that they offered structured follow-up (67–71%) (Table II).

**Table II:** Provided rehabilitation interventions and differences between the settings (*n* = 148)

Rehabilitation interventions and priorities according to the national guidelines for stroke care		Stroke unit (*n* = 28) *n* (%)	Hospital in- and out-patient rehabilitation (*n* = 51) *n* (%)	Primary care (*n* = 30) *n* (%)	Municipal care (*n* = 39) *n* (%)	*p*-value*
Early supported discharge (ESD) Priority 2	Always/frequently provided	19 (68)	6^1^ (100)	Intervention not relevant	Intervention not relevant	
	Rarely/not provided	7 (25)	0 (0)			
	Not relevant in my clinical setting	2 (7)	0 (0)			
Prevention of falls Priority 2	Always/frequently provided	24 (86)	47 (92)	22 (73)	35 (90)	0.433
	Rarely/not provided	3 (11)	1 (2)	1 (3)	3 (8)	
	Not relevant in my clinical setting	1 (4)	3 (6)	7 (23)	1 (3)	
Aerobic and strength training Priority 3	Always/frequently provided	11 (39)	40 (78)	28 (93)	31 (80)	**< 0.001** ^a–c^
	Rarely/not provided	9 (32)	6 (12)	1 (3)	7 (18)	
	Not relevant in my clinical setting	8 (29)	5 (10)	1 (3)	1 (3)	
Task-specific gait training Priority 3	Always/frequently provided	24 (86)	50 (98)	24 (80)	36 (92)	0.070
	Rarely/not provided	2 (7)	1 (2)	4 (13)	3 (7)	
	Not relevant in my clinical setting	2 (7)	0 (0)	2 (7)	0 (0)	
Treadmill training with bodyweight support Priority 6	Always/frequently provided	0 (0)	6 (12)	2 (7)	1 (3)	**0.011** ^a,d^
	Rarely/not provided	17 (61)	38 (75)	16 (53)	26 (67)	
	Not relevant in my clinical setting	11 (40)	7 (14)	12 (40)	12 (31)	
Mirror therapy Priority 4	Always/frequently provided	2 (7)	5 (10)	6 (20)	1 (3)	0.093
	Rarely/not provided	20 (71)	41 (80)	18 (60)	31 (80)	
	Not relevant in my clinical setting	6 (21)	5 (10)	6 (20)	7 (18)	
Constraint-induced movement therapy (CIMT) Priority 4	Always/frequently provided	0 (0)	9 (18)	0 (0)	0 (0)	**0.036** ^a,d,e^
	Rarely/not provided	18 (64)	35 (69)	14 (47)	33 (85)	
	Not relevant in my clinical setting	10 (36)	7 (14)	16 (53)	6 (15)	
Task-specific training for impaired motor function Priority 3	Always/frequently provided	23 (82)	51 (100)	27 (90)	25 (64)	**< 0.001** ^d^
	Rarely/not provided	4 (14)	0 (0)	1 (3)	13 (33)	
	Not relevant in my clinical setting	1 (4)	0 (0)	2 (7)	1 (3)	
Botulinum toxin in combination with therapy Priority 4	Always/frequently provided	1 (4)	34 (67)	4 (13)	4 (10)	**0.001** ^a,d,e^
	Rarely/not provided	13 (46)	6 (12)	6 (20)	19 (49)	
	Not relevant in my clinical setting	14 (50)	11 (22)	20 (67)	16 (41)	
Transcutaneous electrical nerve stimula-tion (TENS) to treat shoulder pain Priority 5	Always/frequently provided	2 (7)	10 (20)	8 (27)	7 (18)	0.054
	Rarely/not provided	21 (75)	39 (77)	21 (70)	27 (69)	
	Not relevant in my clinical setting	5 (18)	2 (4)	1 (3)	5 (13)	
Orthoses for the upper limb, to treat hemiplegic shoulder pain Priority 3	Always/frequently provided	26 (93)	32 (63)	8 (27)	29 (74)	**< 0.001** ^a,b^
	Rarely/not provided	2 (7)	18 (35)	13 (43)	9 (23)	
	Not relevant in my clinical setting	0 (0)	1 (2)	9 (30)	1 (3)	
Education for caregivers Priority 3	Always/frequently provided	4 (14)	29 (57)	8 (27)	25 (64)	**0.002** ^a,c^
	Rarely/not provided	18 (64)	22 (43)	17 (57)	13 (33)	
	Not relevant in my clinical setting	6 (21)	0 (0)	5 (17)	1 (3)	
Structured follow-up Priority 2	Always/frequently provided	Intervention not relevant	36 (71)	20 (67)	Intervention not relevant	
	Rarely/not provided		9 (18)	4 (13)		
	Not relevant in my clinical setting		6 (12)	6 (20)		

1 ESD team only included (*n* = 6).

* The Kruskall–Wallis test was performed to do an overall analysis if differences between various settings existed. Thereafter, pairwise post-hoc tests and Bonferroni corrections were performed to analyse between which settings significant differences occurred. Bold *p*-values indicate significance.

Letters denote significant difference between the settings.

a Stroke unit – hospital in- and outpatient rehabilitation.

b Stroke unit – primary care.

c Stroke unit – municipal care.

d Hospital in- and outpatient rehabilitation – municipal care.

e Hospital in- and outpatient rehabilitation – primary care.

### Differences between the settings regarding provided interventions

Significant differences (*p* < 0.05) regarding provided interventions between the various settings were found for 7 out of 13 interventions (Table II). Aerobic and resistance training (priority 3) was provided less in the stroke unit than in other settings. Task-specific training for impaired motor function (priority 3) was provided less in municipal care than in other settings. Orthoses to support the upper limb to reduce shoulder pain (priority 3) were provided to a greater extent in the stroke unit than in primary care and hospital in- and outpatient rehabilitation. Education for caregivers (priority 3) was most frequently provided in municipal care.

Constraint-induced movement therapy (CIMT, priority 4) was provided neither in primary care nor in municipal care, whereas hospital in- and outpatient rehabilitation provided CIMT to a minor extent. Botulinum toxin injections in combination with other rehabilitation interventions (priority 4) was primarily provided in both hospital in- and outpatient rehabilitation (Table II).

### Factors that facilitated implementation of evidence-based interventions

Factors that the PTs agreed facilitated implementation of evidence-based interventions were: that it was considered important to comply with the NGS (93–100%), that they had confidence to perform the recommendations (64–98%), that the recommendations in the NGS were clearly described (77–90%) and that they had sufficient knowledge of the NGS (67–94%) (Table III).

**Table III:** Perceived factors that facilitated the implementation of the National Guidelines for Stroke care (NGS), and differences between the settings (*n* = 148)

Facilitating factors		Stroke unit (*n* = 28) *n* (%)	Hospital in- and out-patient rehabilitation (*n* = 51) *n* (%)	Primary care (*n* = 30) *n* (%)	Municipal care (*n* = 39) *n* (%)	*p*-value*
Consider it to be important to comply with the NGS	Strongly/somewhat agree	26 (93)	51 (100)	28 (93)	38 (97)	0.257
	Somewhat/strongly disagree	2 (7)	0 (0)	2 (7)	1 (3)	
Sufficient knowledge regarding the NGS	Strongly/somewhat agree	25 (89)	48 (94)	21 (70)	26 (67)	**0.002** ^d,e^
	Somewhat/strongly disagree	3 (11)	3 (6)	9 (30)	13 (33)	
Confidence to perform the recommendations	Strongly/somewhat agree	27 (96)	50 (98)	26 (87)	25 (64)	**< 0.001** ^c,d,f^
	Somewhat/strongly disagree	1 (4)	1 (2)	4 (13)	14 (36)	
Engagement by team members to comply with the NGS	Strongly/somewhat agree	20 (71)	48 (94)	23 (77)	20 (51)	**< 0.001** ^d^
	Somewhat/strongly disagree	8 (29)	3 (6)	7 (23)	19 (49)	
Team members have sufficient knowledge of the NGS	Strongly/somewhat agree	20 (71)	46 (90)	20 (68)	17 (44)	**< 0.001** ^d^
	Somewhat/strongly disagree	8 (29)	5 (10)	10 (33)	22 (56)	
Supportive management to comply with the NGS	Strongly/somewhat agree	19(68)	42 (82)	19 (63)	15 (39)	**< 0.001** ^d^
	Somewhat/strongly disagree	9 (32)	9 (18)	11 (37)	24 (62)	
Can influence the way to comply with the NGS	Strongly/somewhat agree	23 (82)	44 (86)	22 (73)	25 (64)	0.080
	Somewhat/strongly disagree	5 (18)	7 (14)	8 (27)	14 (36)	
Clear routines to promote the work of implementing the NGS	Strongly/somewhat agree	18 (64)	38 (75)	15 (50)	9 (23)	**< 0.001** ^c,d^
	Somewhat/strongly disagree	10 (36)	13 (26)	15 (50)	30 (77)	
Clear goals to promote the work of implementing the NGS	Strongly/somewhat agree	14 (50)	33 (65)	13 (43)	11 (28)	**0.007** ^d^
	Somewhat/strongly disagree	14 (50)	18 (35)	17 (57)	28 (72)	
Enough time to comply with the NGS	Strongly/somewhat agree	11 (39)	36 (71)	15 (50)	16 (41)	**0.013** ^a,d^
	Somewhat/strongly disagree	17 (61)	15 (29)	15 (50)	23 (59)	
Access to adequate equipment needed to comply with the NGS	Strongly/somewhat agree	17 (61)	47 (92)	13 (43)	17 (44)	**< 0.001** ^a,d,e^
	Somewhat/strongly disagree	11 (39)	4 (8)	17 (57)	22 (56)	
Access to space needed to comply with the NGS	Strongly/somewhat agree	17 (61)	44 (86)	17 (57)	17 (44)	**< 0.001** ^d,e^
	Somewhat/strongly disagree	11 (39)	7 (14)	13 (43)	22 (56)	
Access to physiotherapist resources needed to comply with the NGS	Strongly/somewhat agree	14 (50)	41 (80)	15 (50)	13 (33)	**< 0.001** ^a,d,e^
	Somewhat/strongly disagree	14 (50)	10 (20)	15 (50)	26 (67)	
Access to continuous education regarding stroke rehabilitation	Strongly/somewhat agree	23 (82)	42 (82)	19 (63)	14 (36)	**< 0.001** ^c,d^
	Somewhat/strongly disagree	5 (18)	9 (18)	11 (37)	25 (64)	
The recommendations in NGS are clearly described	Strongly/somewhat agree	25 (89)	45 (88)	23 (77)	35 (90)	0.372
	Somewhat/strongly disagree	3 (11)	6 (12)	7 (23)	4 (10)	

* The Kruskall–Wallis test was used to analyse differences between the settings. Thereafter, pairwise post-hoc tests and Bonferroni corrections were performed to analyse between which settings significant differences occurred. Bold *p*-values indicate significance.

Letters denote significant difference between the settings.

a Stroke unit – hospital in- and outpatient rehabilitation.

b Stroke unit – primary care.

c Stroke unit – municipal care.

d Hospital in- and outpatient rehabilitation – municipal care.

e Hospital in- and outpatient rehabilitation – primary care.

f Municipal care – primary care.

### Differences between the settings regarding facilitating factors

Significant differences (*p* < 0.05) between the various settings were found for 12 out of 15 statements (Table III). The most significant differences regarding facilitating factors were reported between PTs working in hospitals with inpatient and outpatient rehabilitation and PTs working in municipal care. A majority of PTs working in hospital in- and outpatient rehabilitation agreed that access to adequate equipment and PT resources facilitated the implementation of the NGS, while PTs working in the other settings agreed to a much lesser extent. Significantly fewer PTs in municipal care agreed with the statement, for example that they had clear routines, sufficient knowledge, access to continued education and supportive management to implement the NGS (Table III).

### Factors that hindered implementation of evidence-based interventions

Factors that PTs agreed hindered implementation of the evidence-based interventions according to the NGS were: limited time to comply with the NGS (55–77%), lack of PT resources needed to comply with the NGS (51–82%), and no clear goals (53–90%) or routines (45–90%) at the workplace to promote implementation of the guidelines (Table IV).

**Table IV:** Perceived factors that hindered the implementation of the National Guidelines for Stroke care (NGS) and differences between the settings (*n* = 148)

Hindering factors		Stroke unit (*n* = 28) *n* (%)	Hospital in- and out-patient rehabilitation (*n* = 51) *n* (%)	Primary care (*n* = 30) *n* (%)	Municipal care (*n* = 39) *n* (%)	*p*-value*
Consider it to be unimportant to comply with the NGS	Strongly/somewhat agree	1 (4)	4 (8)	4 (13)	6 (15)	0.374
	Somewhat/strongly disagree	27 (96)	47 92)	26 (87)	33 (85)	
Lack of knowledge regarding the NGS	Strongly/somewhat agree	7 (25)	16 (31)	18 (60)	26 (67)	**< 0.001** ^b,c,d^
	Somewhat/strongly disagree	21 (75)	35 (69)	12 (40)	13 (33)	
Lack of confidence to perform the recommendations in NGS	Strongly/somewhat agree	4 (14)	11 (22)	8 (27)	17 (44)	**0.038** ^c^
	Somewhat/strongly disagree	24 (86)	40 (78)	22 (73)	22 (56)	
Lack of engagement by team members to comply with the NGS	Strongly/somewhat agree	8 (29)	10 (20)	7 (23)	19 (49)	**0.021** ^d^
	Somewhat strongly disagree	20 (71)	41 (80)	23 (77)	20 (51)	
Team members have insufficient knowledge of the NGS	Strongly/somewhat agree	10 (36)	22 (43)	17 (57)	29 (74)	**0.006** ^c,d^
	Somewhat/strongly disagree	18 (64)	29 (57)	13 (43)	10 (26)	
Unsupportive management to comply with the NGS	Strongly/somewhat agree	11 (39)	10 (20)	12 (40)	29 (74)	**< 0.001** ^c,d,f^
	Somewhat/strongly disagree	17 (61)	41 (80)	18 (60)	10 (26)	
Cannot influence the way to comply with NGS	Strongly agree/somewhat agree	10 (36)	13 (26)	16 (53)	27 (69)	**< 0.001** ^c,d^
	Somewhat/strongly disagree	18 (64)	38 (75)	14 (47)	12 (31)	
No clear routines to promote the work of implementing the NGS	Strongly/somewhat agree	16 (57)	23 (45)	20 (67)	35 (90)	**< 0.001** ^c,d^
	Somewhat/strongly disagree	12 (43)	28 (55)	10 (33)	4 (10)	
No clear goals to promote the work of implementing the NGS	Strongly/somewhat agree	16 (57)	27 (53)	19 (63)	35 (90)	**0.002** ^c,d^
	Somewhat/strongly disagree	12 (43)	24 (47)	11 (37)	4 (10)	
Limited time to comply with the NGS	Strongly/somewhat agree	21 (75)	28 (55)	21 (70)	30 (77)	0.110
	Somewhat/strongly disagree	7 (25)	23 (45)	9 (30)	9 (23)	
Lack of adequate equipment needed to comply with the NGS	Strongly/somewhat agree	17 (61)	16 (31)	24 (83)	30 (77)	**< 0.001** ^d,e^
	Somewhat/strongly disagree	11 (39)	35 (69)	5 (17)	9 (23)	
Lack of space needed to comply with the NGS	Strongly/somewhat agree	16 (57)	18 (35)	17 (57)	27 (69)	**0.013** ^d^
	Somewhat/strongly disagree	12 (43)	33 (65)	13 (43)	12 (31)	
Lack of physiotherapist resources needed to comply with the NGS	Strongly/somewhat agree	20 (71)	26 (51)	21 (70)	32 (82)	**0.017** ^d^
	Somewhat/strongly disagree	8 (29)	25 (49)	9 (30)	7 (18)	
Lack of access to continuous education regarding stroke rehabilitation	Strongly/somewhat agree	12 (43)	22 (43)	18 (60)	30 (77)	**0.006** ^c,d^
	Somewhat/strongly disagree	16 (57)	29 (57)	12 (40)	9 (23)	
Recommendations in NGS are not clearly described	Strongly/somewhat agree	8 (29)	21 (41)	15 (50)	13 (33)	0.332
	Somewhat/strongly disagree	20 (71)	30 (59)	15 (50)	26 (67)	

* The Kruskall–Wallis test was used to analyse whether differences between settings existed. Thereafter, pairwise post-hoc tests and Bonferroni corrections were performed to analyse between which settings, significant differences occurred. Bold *p*-values indicate significance.

Letters denote significant difference between the settings:

a Stroke unit – hospital in- and outpatient rehabilitation.

b Stroke unit – primary care.

c Stroke unit – municipal care.

d Hospital in- and outpatient rehabilitation – municipal care.

e Hospital in- and outpatient rehabilitation – primary care.

f Municipal care – primary care.

### Differences between the settings regarding hindering factors

Significant differences (*p* < 0.05) between the various settings were found for 12 out 15 statements (see Table IV). The most significant differences regarding hindering factors were reported between PTs working in municipal care and PTs working in hospital in- and outpatient rehabilitation and in stroke units. A majority of PTs working in municipal care agreed that team members had insufficient knowledge of and education on the NGS, unsupportive management to comply with the NGS, were not able to influence the way to comply with the NGS, and had no clear goals and routines to promote the work of implementing the NGS, while PTs working in other settings agreed to a lesser extent. Also, PTs working in primary care agreed that lack of knowledge regarding the NGS and lack of equipment hindered the implementation of the guidelines compared with the other settings.

## DISCUSSION

The results in the present study indicate that most evidence-based interventions with high priority according to the NGS were provided to a great extent by PTs working in stroke rehabilitation in various settings in Sweden, although differences existed. The main factors that participants agreed facilitated implementation of the interventions were that it was important to comply with the NGS, that they had confidence to perform the interventions, that they were clearly described and that the PTs had sufficient knowledge of the NGS. The main factors that participants agreed hindered the implementation were limited time, lack of PT resources and no clear goals or routines at the workplace to promote implementation of the NGS. Regarding differences between settings, PTs working in municipal and primary care reported most challenges in implementing the NGS and providing recommended interventions.

Regarding implementation of evidence-based interventions in various settings, we found that many PTs working at a stroke unit always/frequently provide ESD and continued rehabilitation at home. It is shown that ESD can lead to a reduction in long-term dependency and reduce the length of hospital stay (26) and therefore this figure is pleasing. This indicates that ESD is provided to a greater extent compared with 2018, when the Swedish stroke register reported that many hospitals in Sweden could not provide ESD (7), and that the Swedish National Board of Health and Welfare’s goal that ≥ 25% of all stroke units should provide ESD may be achieved (27).

Another intervention that has high priority, according to the NGS, is structured multidisciplinary follow-up post-stroke (1). Our findings indicate that a majority of the PTs working in in- and outpatient rehabilitation and in primary care provided structured follow-up. However, a previous study has shown that 25% of individuals post-stroke are not followed up within three months after hospital discharge (28). Tistad et al. (29) have reported that people with moderate to severe stroke who had seen a PT at least once during the first year post-stroke were more likely to report met rehabilitation needs. Thus, continuity in rehabilitation and reassessment by a multidisciplinary team during the first year after stroke is important and could contribute to meeting the rehabilitation needs after stroke.

Furthermore, it is shown that impaired hand function can predict the patient’s perceived unfulfilled rehabilitation need one year after stroke onset (30). CIMT was developed to overcome disability of the upper limb after stroke and is one of the most investigated interventions during the last decades (31). The NGS recommends that CIMT (in terms of modified CIMT) can be provided after stroke (1). Currently, however, mCIMT/CIMT is provided only in some clinics in Sweden (1), which is in line with the results of the present study. The reason for this may be that mCIMT/CIMT is too resource consuming, although the training protocol varies with regard to intensity and duration (1). Furthermore, mirror therapy was also provided to a lesser extent in all settings (Table II). This was somewhat surprising, as the intervention has priority 4 in the NGS and can be sufficient for those with moderate to severe impairments in the upper extremity after stroke (1).

Treatment with botulinum toxin in combination with other rehabilitation interventions to reduce spasticity can also be provided after stroke (1). This intervention was provided only in hospital in- and outpatient rehabilitation, and seldom in primary care and municipal care. Our result is in agreement with a previous Swedish study reporting inequalities and regional differences in treatment with botulinum toxin of spasticity in adults (32). The results can be explained by the fact that the distribution of team-based spasticity clinics is unevenly distributed in Sweden (1).

Regarding perceived factors that facilitate the implementation process of evidence-based interventions, the results from the present study confirm previous research, showing that training in how to use the guidelines and increased resources is important (13, 33). Jucket et al. reported in their review (25) that hindering factors in the implementation process are lack of resources and adequate equipment, as well as lack of knowledge concerning the interventions. Facilitating factors were good knowledge of the interventions and clear support and commitment from management. To have access to resource personnel with good knowledge of evidence-based practice was also supportive (25). Several of these factors were also reported in our study and are important to consider in all healthcare settings, in order to facilitate care and rehabilitation after stroke becoming more equal. How-ever, our findings indicate that there are differences regarding factors that PTs agreed facilitated/hindered the implementation of the NGS, especially between PTs working in municipal care and those working in hospital in- and outpatient rehabilitation and in stroke units. This underscores the importance of managers taking relevant measures, adapted to local practice needs, to further facilitate the implementation process in their own clinical setting (12).

Previous research has shown that in order to promote implementation of NGS it is important that the PTs have sufficient knowledge regarding stroke rehabilitation and the guidelines (13, 25, 33). The results from the present study show that PTs working in municipal and primary care reported that insufficient knowledge hindered implementation of the NGS. Our results support the importance of offering PTs and other team members education regarding evidence-based interventions, so they feel confident to provide them (14, 15, 25). The National Board of Health and Welfare’s latest report on the evaluation of stroke care (34) shows that a minority of healthcare professionals working in municipal care have further education in stroke rehabilitation. Continuous training support is therefore of great importance in order to develop competencies and an efficient teamwork.

Just over half of the respondents in our study reported that they had further education in stroke rehabilitation and 9% that they had specialist competence in neurology. Currently, there are only 150 PTs with specialist competence in neurology in Sweden, according to the Swedish Association for Physiotherapists (28 February 2022) (35). It would be desirable for there to be more PTs with specialist expertise in neurology at every level of the care chain in Sweden, as they could be a local clinical facilitator and contribute by improving the implementation processes of evidence-based practice (11, 12, 15). But, overall, the role of context is of importance for successful implementation of evidence-based interventions, and there is a need for continuous support for PTs and other stroke care professionals, from managers and other decision-makers in various care settings, to advocate and support the use of evidence-based interventions (13).

### Methodological considerations

A strength of this study is that the questions in the survey were inspired by the theoretical framework, the PARIHS model, including questions on evidence-based interventions, context (settings) and facilitating and hindering factors for implementation. To ensure that the questions in the survey were not misinterpreted, a pilot survey was first distributed to PTs working at various levels of the stroke care chain. Based on their comments, slight adjustments were made, which resulted in the final version. The survey was directed to PTs working in stroke rehabilitation at hospitals, primary and municipal care. PTs from all 21 counties responded to the survey but only from 19 of 290 municipalities. More respondents from the municipalities would have been desirable, in order to strengthen the generalizability of the results. Also, there is a risk that the PTs who have responded are those who have a positive attitude towards the guidelines. However, the use of a digital survey made it possible to reach many PTs in different care settings across Sweden. One limitation of such a questionnaire is that it is based on predefined questions and that the results are a reflection on that. The participants were not able to ask follow-up questions, and thus there is a risk that the responses may be influenced by misinterpretations of the questions (36). Bearing in mind that there are some limitations with this study, the results should be interpreted with caution.

### Clinical implications

The result of the present study contributes to increased knowledge concerning which evidence-based interventions are most provided at various levels of the stroke care chain in Sweden, as well as knowledge of differences in the various settings regarding factors that PTs agreed facilitated/hindered the implementation process. By pointing out which interventions are not provided, managers at various levels of the stroke care chain can take relevant measures to further facilitate the implementation process in their own settings (33). Future studies should include other stroke care professionals, and investigate to what extent they have implemented other interventions according to the NSG, and whether national differences do exist.

In conclusion, the result of the present study indicates that most interventions with high priority according to the NGS are provided by PTs working in stroke rehabilitation, although differences in various parts of the care chain exist. Knowledge, time, education and supportive management are important factors when implementing evidence-based interventions. Municipal and primary care seem to have most challenges in implementing the NGS and providing evidence-based interventions.
